# Human intravital microscopy in the study of sarcomas: an early trial of feasibility

**DOI:** 10.3389/fonc.2023.1151255

**Published:** 2023-04-12

**Authors:** Emmanuel M. Gabriel, Kulkaew Sukniam, Kyle Popp, Sanjay P. Bagaria

**Affiliations:** ^1^ Department of General Surgery, Division of Surgical Oncology, Mayo Clinic, Jacksonville, FL, United States; ^2^ Department of General Surgery, Philadelphia College of Osteopathic Medicine, Suwanee, GA, United States; ^3^ Florida State University, Tallahassee, FL, United States

**Keywords:** sarcoma, vessels, imaging, intravital, clinical trial

## Abstract

Sarcomas comprise a vast and heterogenous group of rare tumors. Because of their diversity, it is challenging to study sarcomas as a whole with regard to their biological and molecular characteristics. This diverse set of tumors may also possess differences related to their tumor-associated vasculature, which in turn may impact the ability to deliver systemic therapies (e.g., chemotherapy, targeted therapies, and immunotherapy). Consequently, response to systemic treatment may also be variable as these depend on the ability of the therapy to reach the tumor target *via* the tumor-associated vasculature. There is a paucity of data regarding sarcoma-related tumor vessels, likely in part to the rarity and heterogeneity of this cancer as well as the previously limited ability to image tumor-associated vessels in real time. Our group has previously utilized confocal fluorescent imaging technology to observe and characterize tumor-associated vessels in real time during surgical resection of tumors, including cutaneous melanoma and carcinomatosis implants derived from gastrointestinal, gynecological, or primary peritoneal (e.g., mesothelioma) tumors. Our prior studies have demonstrated the feasibility of real-time, human intravital microscopy in the study of these tumor types, leading to early but important new data regarding tumor vessel characteristics and their potential implications on drug delivery and efficacy. In this brief report, we present our latest descriptive findings in a cohort of patients with sarcoma who underwent surgical resection and real-time, intravital microscopy of their tumors. Overall, intravital imaging was feasible during the surgical resection of large sarcomas.

**Clinical trial registrations:**

ClinicalTrials.gov, identifier NCT03517852; ClinicalTrials.gov, identifier NCT03823144.

## Introduction

Sarcomas comprise a vast, heterogeneously diverse group of malignancies that afflict connective tissues of the body, including bone, nerve, and soft tissue. There are over 80 defined sarcomas, each with specific therapeutic approaches that are often multidisciplinary and require a high level of expertise to provide the most optimal outcome (1, 2). Despite the diversity of this group of tumors, even the most common types of sarcomas are relatively rare compared to other cancers (such as cutaneous tumors or breast cancer). This leads to inherent difficulty in investigating the underlying molecular biology and biodiversity of this group of tumors, and likely contributes to the limited sarcoma treatment options compared to other cancers, resulting in high rates of tumor recurrence or disease progression (3). Similar to these challenges, the tumor-associated vasculature of sarcomas has largely been unstudied. While certain sarcomas by their nature are intrinsically vascular (namely angiosarcomas), the extent of vascularity for many of the more common sarcomas (such as gastrointestinal stromal tumors or GISTs, liposarcomas, and leiomyosarcomas) are undefined.

Our group has utilized innovative technologies to study tumor-associated vessels in real time among patients undergoing surgical resection (4). These investigations were initially performed in patients undergoing wide local excision of cutaneous melanoma (5). In this first trial of its kind, real-time, intravital fluorescence microscopy was successfully performed to identify, characterize, and quantify melanoma-associated vessels, revealing drastic differences in human tumor-associated vessels as compared with normal (control) vessels. We have since broadened our study of tumor-associated vessels by using human intravital microscopy to examine the tumor vessels in patients with peritoneal carcinomatosis (6). In this recent clinical trial, further differences in tumor vessel density and functionality were identified, whereby patients who had received neoadjuvant systemic therapies and experienced partial response qualitatively had lower proportions of non-functional tumor-associated vessels and higher proportions of functional, normal vessels compared to patients who had stable or progressive disease. Other groups have also started to investigate tumor-associated vessels in other cancer settings in humans, including esophagogastric, colorectal, and bladder cancers (7–10). These differences are important to recognize and define because systemic drug delivery is highly dependent on the tumor vasculature (11–13). Our group and others have shown that manipulation or optimization of blood flow through tumor-associated vessels at the time of drug delivery can improve tumor responses in animal cancer models (14–17). However, little is known about these interventions on sarcoma-associated vessels. Therefore, to increase the understanding of sarcoma tumor-associated vessels, we analyzed a cohort of 10 sarcoma patients who underwent surgical resection and real-time, intraoperative tumor vessel imaging. Our main objective was to determine the feasibility of intravital microscopy (IVM) in the observation of sarcoma-related tumor vessels. Herein, we present a descriptive analysis of our results.

## Patients and methods

### Patient selection

Patients were enrolled in single center, nonrandomized clinical trials at Mayo Clinic in Jacksonville, Florida, USA. These trials included Intravital Microscopy (IVM) in Patients with Peritoneal Carcinomatosis (ClinicalTrials.gov identifier: NCT03517852) and Intravital Microscopy in Human Solid Tumors (ClinicalTrials.gov identifier: NCT03823144) (6, 8). Both trials received IRB approval from the Mayo Clinic (IRB #17-009823 and IRB #18-010370, respectively). The protocols for these trials, and the inclusion and exclusion criteria had previously been reported and are available through nct.gov (6, 8). Briefly, patients underwent informed consent for participation and received a fluorescein skin prick test to evaluate the low risk of an allergic reaction to the fluorescein dye used to enhance the intravital in-human observations of blood vessels. Recorded information included demographic data (age, sex, body-mass index, race, history of smoking, history of diabetes, prior abdominal surgery), sarcoma-specific data (tumor histology and subtype, grade, primary versus recurrent diagnosis, size of the primary tumor with the longest dimension reported), and treatment-related variables (receipt and type of neoadjuvant and/or adjuvant chemotherapy or other systemic therapy; receipt of neoadjuvant radiotherapy, radiographic response to neoadjuvant therapy as measured by standard RECIST criteria, surgical approach, and complications from surgery including cytoreduction surgery with or without hyperthermic intraperitoneal chemotherapy, or CRS-HIPEC, when applicable).

### Intravital microscopic observations in patients

The technique for real-time human intravital microscopy had also been previously described by our group (6, 8). Briefly, we utilized the ultra-high definition (UHD) probe-based confocal laser endomicroscopy device (Gastroflex, Cellvizio System, Mauna Kea Technologies, Paris, France). Sarcoma-associated vessels were observed at 100x magnification. Videos were obtained in a proprietary format video files (.mkt) for *post hoc* data analysis. Offline quantification of vessel characteristics was performed using the Mauna Kea Technologies IC-Viewer (Mauna Kea Technologies, Paris, France). All images/videos were stored on a password-protected institutional hard drive for later analysis.

Prior to surgical resection, tumor vessel observations were performed on two separate sarcoma-bearing areas and two separate non-tumor bearing control areas. The tumor areas and control areas were separated by a distance of at least 10 centimeters (cm). We selected the tumor areas based on the highest amount of gross tumor burden that was visualized, which was up to the surgeon’s discretion at the time of the resection. Common gross characteristics of abnormal tissue included color (often white or gray compared to yellow normal tissue), texture (often firm compared to soft), and infiltration into normal organs or structures. Observations occurred either through open or minimally invasive (laparoscopic or robotic) approaches. Prior to the observation, the surgeon instructed the anesthesiologist to administer 1 ml of fluorescein (AK-Fluor^®^ fluorescein, 10% at 100 mg/mL) intravenously followed by a 10 ml saline flush. During the fluorescein administration, the Gastroflex probe was positioned over the first predetermined sarcoma field to be ready for observation once the recording was initiated. After 10-15 seconds of administering the fluorescein, the dye could be visualized within functional tumor vessels. Each predetermined area was observed for 60-90 seconds, for a total of about 4-6 minutes for the entire patient observation. Within each area, multiple fields were observed over an area of approximately 2 square cm during each observation period. In order to facilitate stabilization of the HIVM observations, respirations were temporarily restricted by the anesthesiologist for a maximum of approximately 30 seconds per observed area. By approximately 5-6 minutes after the fluorescein administration, the dye (which has a molecular weight of 332.31 g/mol) extravasated into the background stroma, which in turn increased the fluorescent signal of the surrounding stromal tissue as compared to any functional vessels (normal or tumor-associated) present within the field of view. Similar to our prior study in peritoneal carcinomatosis, the observation for a given patient was completed when fluorescein was noted to have extravasated out of tumor/control vessels into the surrounding background tissue (6).

Characterization of the tumor vessels and the measured parameters had previously been described by our group (5, 6, 9). Briefly, we characterized the following tumor vessel characteristics during the intraoperative observations: (1) vessel identification per high power field, (2) vessel density, (3) fluorescein uptake as a measure of tumor vessel functionality (dye uptake) or non-functionality (lack of dye uptake), and (4) blood flow velocity. Vessel density (for both functional and non-functional) was calculated by dividing the number of vessels by the number of fields of observation per area (control or tumor). The percentage of non-functional vessels per area (tumor or control) was calculated by dividing the number of non-functional vessels by the total number of vessels observed (# non-functional vessels/# non-functional vessels + # functional vessels) times 100. IC-Viewer software was used to measure vessel diameter (d) at the vessel’s largest width as well as vessel length prior to any branching points. Blood flow velocity (v) was evaluated by determining the time that distinct features (e.g., a prominent red blood cell or clump of red blood cells) in an observed vessel would take to travel a known distance. Velocity was calculated by dividing the measured distance by the time taken to travel that distance, and then averaging these values for at least 3 points per vessel. No velocities were calculated for non-functional vessels as by definition, these vessels did not support any blood flow.

### Statistical analyses

Demographic and clinical characteristics were summarized using the mean and standard deviation (std) for continuous variables, and using frequencies for categorical variables. Vessel characteristics (diameter, density, and velocity) were summarized using mean and std. The two-sided, paired t test was used to make comparisons between the control and treatment groups. Progression-free (PFS) and disease-specific survival (DSS) were summarized using standard Kaplan-Meier methods. All analyses were conducted in SAS v9.4 (Cary, NC) at a significance level of 0.05. As this was a study of IVM feasibility, the statistical analysis was limited to descriptive statistics. Correlative analyses of vessel characteristics with outcomes (response to neoadjuvant chemotherapy, PFS, or DSS) were not performed.

## Results

### Patient demographics, tumor-specific characteristics, and treatment outcomes

Between January 1, 2018 and December 31, 2022, we enrolled 10 patients with sarcoma as part of our clinical trials (NCT03517852 Intravital Microscopy in Patients with Peritoneal Carcinomatosis and NCT03823144 Intravital Microscopy in Human Solid Tumors). Patient and tumor-specific characteristics are shown in **Table 1**. Most patients had retroperitoneal liposarcomas (5 total, 2 well-differentiated and 3 dedifferentiated). Half of the tumors (5/10) were high grade, and 4 were recurrences. Only 2 patients received neoadjuvant systemic chemotherapy (including doxorubicin/ifosfamide/mesna and doxorubicin/olaratumab), but 4 patients received neoadjuvant radiation (55-60 Grey). Of these patients, there were no partial responses, and 3 patients had stable disease with 1 patient having disease progression. One patient received multiple rounds of adjuvant chemotherapy (including gemcitabine/docetaxel, doxorubicin/olaratumab, and pazopanib). One patient also required liver ablation for a metastasis. Two patients had died at the time of our analysis. The individual progression-free and disease-specific survivals are reported in **Table 1**. The median follow-up was 2.5 years. Unlike our most recent study in peritoneal carcinomatosis, correlations of tumor vessel characteristics and response to neoadjuvant treatment or survival outcomes were not calculated due to the small cohort of sarcoma patients.

**Table 1:** Patient, tumor, and treatment-related variables.

Variable		N (#)
Age (years)	mean (std)	62.1 (10.8)
Sex	female	3
	male	7
Body mass index (BMI)	mean (std)	30.5 (2.6)
Race	Asian	1
	White	9
Smoking history	current	0
	former	1
	never	9
Diabetes	no	8
	yes	2
Histology	retroperitoneal liposarcoma	5
	gastrointestinal stromal tumor	2
	uterine leiomyosarcoma	1
	pleomorphic sarcoma	1
	leiomyosarcoma	1
Grade	low/well-differentiated	4
	intermediate	1
	high/dedifferentiated	5
Recurrence	no	4
	yes	6
Tumor size (longest dimension, cm)	mean (std)	8.8 (5.5)
Previous abdominal surgery	no	4
	yes	6
Surgical approach	open	8
	laparoscopic/robotic	2
Neoadjuvant therapy	no	8
	yes	2
Neoadjuvant radiotherapy	no	6
	yes	4
Adjuvant chemotherapy	no	9
	yes	1
RECIST response (N = 4)	partial response	0
	stable disease	3
	progressive disease	1
Survival (median months, 95% CI)	progression-free	2.6 (2.5, 2.7)
	disease-specific	2.9 (2.8, 3.0)

### HIVM vessel characteristics

**Table 2** shows the vessel characteristics obtained from the in-human intravital observations. Similar numbers of tumor and control fields were observed within our patient cohort (p = 0.22). Statistically significant differences between the tumor and control fields were observed for some blood vessel characteristics. Similar to our previous study in peritoneal carcinomatosis, tumor-associated areas were observed to have a higher density of non-functional vessels (p < 0.0018) and a higher proportion of non-functional vessels compared to non-tumor control areas (p < 0.0032) (6). Similarly, the mean blood flow velocity of functional vessels within tumor areas was significantly slower than the mean velocity of functional vessels within non-tumor areas (p < 0.0001), which was also the case with the tumor-associated vessels in patients with carcinomatosis.

**Table 2:** Comparison of tumor and non-tumor (control) vessel characteristics during intravital microscopic observations.

Variable		Sarcoma Cohort (N = 10)	
		Mean (Std, Range)	P value
Number of observed fields	Control	6.67 (1.94, 3-12)	0.22
	Tumor	8.20 (2.96, 4-10)	
Density of functional vessels	Control	2.12 (0.88)	0.92
	Tumor	2.17 (1.22)	
Density of non-functional vessels	Control	0.11 (0.17)	0.0018
	Tumor	1.02 (0.72)	
% Non-functional vessels	Control	4.59 (7.68)	0.0032
	Tumor	35.69 (26.18)	
Diameter of functional vessels (μm)	Control	23.35 (12.5)	0.35
	Tumor	18.05 (11.36)	
Diameter of non-functional vessels (μm)	Control	14.11 (3.27)	0.034
	Tumor	21.34 (8.86)	
Length of functional vessels (μm)	Control	187.55 (52.64)	0.78
	Tumor	166.72 (68.72)	
Length of non-functional vessels (μm)	Control	148.83 (78.41)	0.67
	Tumor	160.29 (49.65)	
Velocity of functional vessels (μm/s)	Control	430.49 (49.74)	<0.0001
	Tumor	286.77 (44.45)	

Conversely, there were no statistically significant differences between the density of functional vessels (p = 0.92), the diameter of functional vessels (p = 0.35) within control and tumor areas, or the vessel length among either functional (p = 0.78) or non-functional (p = 0.67) vessels within control and tumor areas. Some of these findings were different from our study in peritoneal carcinomatosis, where there were statistically significant differences among these parameters. Specifically, there was a lower density of functional vessels associated with carcinomatosis, and the average diameter of functional vessels was smaller in tumor areas compared with the diameter of functional vessels in control areas (6). In our prior study in patients with peritoneal carcinomatosis, the mean diameter of non-functional vessels was similar between the tumor and non-tumor areas (p = 0.15). However, in this study of patients with sarcoma, there was a statistically significant difference in the diameter of non-functional vessels, whereby non-functional tumor vessels were larger than non-functional control vessels. **Table 3** summarizes the differences between vessel characteristics in our current study with sarcoma and our previous study in patients with peritoneal carcinomatosis (NCT03517852 Intravital Microscopy in Patients with Peritoneal Carcinomatosis).

**Table 3:** Comparison of vessel characteristics between sarcoma (current trial) and peritoneal carcinomatosis (previous trial NCT03517852 Intravital Microscopy in Patients with Peritoneal Carcinomatosis).

Variable	Sarcoma	Peritoneal Carcinomatosis
	control versus tumor	
Number of observed fields	No difference	No difference
Density of functional vessels	No difference	Control > tumor
Density of non-functional vessels	Tumor > control	Tumor > control
% Non-functional vessels	Tumor > control	Tumor > control
Diameter of functional vessels (μm)	No difference	Control > tumor
Diameter of non-functional vessels (μm)	Tumor > control	No difference
Velocity of functional vessels (μm/s)	Control > tumor	Control > tumor

**Figure 1:**
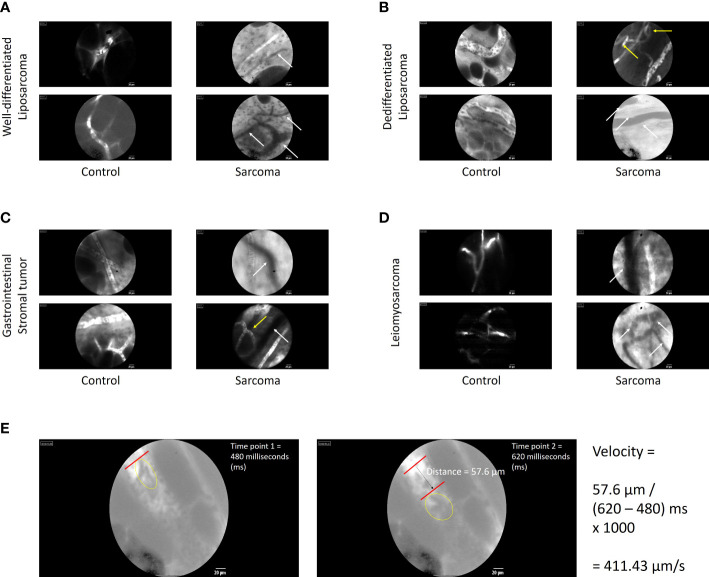
Images of tumor and non-tumor vessels among individual patients with sarcoma. Observations of sarcoma-associated vessels from well-differentiated **(A)** and dedifferentiated **(B)** retroperitoneal liposarcomas, gastrointestinal tumors **(C)**, and leiomyosarcomas **(D)** are depicted for both control and sarcoma areas. Similar to our previous clinical trials in melanoma and peritoneal carcinomatosis, aberrant and non-functional sarcoma-associated vessels could be observed using our intravital microscope. Arrows highlight qualitative differences between normal and sarcoma-associated vessels. White arrows highlight nonfunctional vessels (absence of fluorescent dye uptake). Yellow arrows indicate aberrant loop or branch patterns. **(E)** Example calculation of how blood flow velocity was observed and estimated by tracking a distinct cluster of red blood cells traveling through a functional blood vessel in a visualized normal (control) area for a patient with a dedifferentiated liposarcoma. The left frame shows the cluster of interest (within yellow oval) at first time point. The right frame shows the same cluster traveling a measurable distance with the blood vessel. The velocity calculation is shown to derive velocity in μm/s.

Representative examples of real-time images of sarcoma and non-tumor vessels among individual patients are shown in **Figure 1** (scale bar = 20 μm). Observations from well-differentiated (A) and dedifferentiated (B) retroperitoneal liposarcomas, gastrointestinal tumors (C), and leiomyosarcomas (D) are depicted for both control and sarcoma areas. The outlines of individual adipocytes could be visualized within the normal fatty area controls in the liposarcoma panels (A and B). Individual red blood cells could also be observed within a given functional blood vessel. Part E demonstrates an example of how blood flow velocity was observed and estimated by tracking a distinct cluster of red blood cells traveling through a functional blood vessel in a visualized normal (control) area for a patient with a dedifferentiated liposarcoma. Similar to our previous clinical trials in melanoma and peritoneal carcinomatosis, aberrant and non-functional sarcoma-associated vessels could be observed using our intravital microscope. There was a high proportion of non-functional vessels observed in tumor areas, with several panels demonstrating the juxtaposition of functional and non-functional vessels within the same field of view. Arrows highlight qualitative differences between normal and sarcoma-associated vessels.

## Discussion

To our knowledge and extensive review of the literature, this was the first in-human study of sarcoma-associated vessels in real time. When comparing sarcoma-associated vessels to normal control vessels, we expectedly demonstrated that tumor areas had on average a higher density and proportion of non-functional vessels compared to control areas. This was observed in our previous trials in melanoma and peritoneal carcinomatosis (5, 6). However, unlike our previous studies, in this trial we demonstrated differences among other vessel characteristics, specifically among the density of functional vessels, the diameter of functional vessels, and the diameter of non-functional vessels (**Table 3**). Interestingly, these differences are consistent with the increasing body of knowledge that supports a vascular heterogeneity among tumor types. Indeed, there is considerable tumor vessel heterogeneity (in addition to tumor genetic and molecular heterogeneity) that exists between tumor types. While most of this data has been reported in animal models, there are some clinical studies that describe tumor vessel heterogeneity. For example, Mezheyeuski et al. showed in pathologic stage II/III colon cancer specimens (312 patients) that increased vessel density was associated with increased response to chemotherapy (18). Also pertaining to colorectal cancer, Herrera et al. showed that tumor-associated vessels may influence the interaction between stromal fibroblasts and circulating effector T cells, potentially optimizing immune crosstalk and anti-tumor responses (19). Using preclinical models of melanoma, our own group has demonstrated that drug delivery can depend on intra-arterial or intravenous delivery, and that blood flow through tumor-associated vessels can be optimized to enhance systemic treatment delivery, which in turn improves therapeutic responses (16, 17). Our ongoing translational studies seek to develop methods of dynamic tumor vessel control with the end goal of optimizing tumor blood flow at the time of systemic drug delivery. These include experiments with fluorescently-labeled effector T cells, fluorescently-labeled liposomal nanoparticle formulations of sustained-released targeted therapies, and chemotherapies that display intrinsic autofluorescence (e.g., doxorubicin), each of which are combined with dynamic tumor vessel control. Furthermore, other groups have shown that modulation of tumor vessels through targeted nanoparticle technology can normalize blood flow through neurologic tumors, leading to increased drug delivery with the same goal of improving treatment response (20, 21).

These findings from neurologic cancers, gastrointestinal cancers, and cutaneous malignancies are imperative to more comprehensively understand and investigate because tumor-associated vessel heterogeneity (with regard to structure and functionality) may impact drug delivery and therefore affect multidisciplinary treatment efficacy (22–25). It has been increasingly shown that the most successful chemotherapeutic agents and targeted inhibitors require adequate distribution to the tumor *via* the circulation, or else they are rendered ineffective (14, 26–29). Even cell-based onco-immunotherapeutics, such as adoptive cell transfer and CAR T cells, are similarly dependent upon accessing the tumor *via* the vasculature, and better outcomes have been directly correlated to immune cell infiltration of the tumor (30–32). A considerable amount of study has focused on elucidating the properties of tumor vessels and overcoming limitations to therapy that involve these heterogeneously organized and functional vessels (33–37). Therefore, our trial provides the initial step in characterizing vessel heterogeneity among patients with sarcoma, which are often refractory to radiation and systemic treatments.

Key to our IVM trial was the use of the Gastroflex fluorescent microscope. IVM provides several advantages over conventional imaging techniques, such as ultrasound, computerized tomography (CT) scan, or magnetic resonance imaging (MRI). Whereas these more conventional imaging techniques provide a more global view of these often large sarcomas, they cannot provide imaging resolution at the capillary level in the way that IVM can (as shown in **Figure 1**) (9). IVM can also provide longer real-time imaging at the time of surgery, where differences in blood pressure or blood flow can be analyzed. However, currently IVM is mostly limited to imaging of surface malignancies. In our current report, only the surface of the sarcomas could be observed and imaged. More conventional techniques (US, CT, MRI) can better characterize the inner portions of the tumor, which often display heterogeneous characteristics compared to the superficial tumor areas. For example, the tumor surface tends to be the most active in terms of replication and growth as the tumor expands outwards and display increased enhancement on contrasted imaging modalities, whereas the core of the tumor tends to become more necrotic as the vasculature cannot support the deeper tumor tissues and so may display decreased internal enhancement. Thus, there are limitations to using IVM to analyze parenchymal tumors, unless they are exposed during surgical resection. Indeed, at this time IVM may be very useful for surface malignancies such as cutaneous cancers as our group previously demonstrated (5). In addition, during resection of larger sarcomas (greater than 10 cm), we were limited in observing the most grossly abnormal tumor areas. While observations of the entire sarcoma could be performed during the course of the surgery, this would have significantly prolonged operative times and exposure to anesthesia, which may have increased the undue risks for patients. Therefore, while we assume that there would be similarities to the tumor vasculature along different surface areas of the sarcoma, additional sarcoma-related vessel heterogeneity may exist that could not be characterized by our approach.

We recognize that there are other limitations in our analysis, similar to our previous studies (5, 6). Our cohort of sarcoma patients was small and only one-third of the patients in our previous trial of peritoneal carcinomatosis. Consequently, this brief research report focuses mainly on the feasibility of IVM in the study of sarcomas and only provides a descriptive analysis of the results. In addition, there was considerable diversity among the tumor histologies within our sarcoma patient cohort. Therefore, this trial was not sufficiently powered to analyze any associations between tumor-associated vessel characteristics and outcomes, namely response to neoadjuvant therapies and survival (PFS and DSS). However, our continuation of NCT03823144 Intravital Microscopy in Human Solid Tumors will likely address this limitation of statistical power with a larger cohort of patients. In fact, to date we have enrolled 30 patients with ovarian tumors who have received neoadjuvant chemotherapy. Our future investigation will evaluate tumor vessel characteristics with response to neoadjuvant chemotherapy and survival outcomes.

In conclusion, despite the acknowledged limitations, this analysis represents the first in-human study of sarcoma-associated vessels in real time. While similarities were identified among sarcoma-associated vessels and peritoneal carcinomatosis-associated vessels from our previous trial, there were also significant differences providing new, direct real-time evidence to support the existence of vascular heterogeneity for different tumors and the feasibility to observe these differences during the course of surgical resection. Further study with a larger cohort of patients with a single type of cancer will potentially correlate tumor-associated vessels with treatment outcomes and help tailor individualized anti-cancer therapy.

## Data availability statement

The raw data supporting the conclusions of this article will be made available by the authors, without undue reservation.

## Ethics statement

The studies involving human participants were reviewed and approved by Mayo Clinic IRB. The patients/participants provided their written informed consent to participate in this study.

## Author contributions

EG and SB developed the concept, protocol, and IRB approval. EG and SB enrolled patients. EG analyzed the data. KS and KP organized the data and edited the manuscript. Each author drafted and approved the final version.
